# Surface Dissolution UV Imaging for Investigation of Dissolution of Poorly Soluble Drugs and Their Amorphous Formulation

**DOI:** 10.1208/s12249-019-1317-z

**Published:** 2019-02-13

**Authors:** Chiau Ming Long, Kin Tang, Hitesh Chokshi, Nikoletta Fotaki

**Affiliations:** 1 Department of Pharmacy and Pharmacology, University of Bath, Claverton Down, Bath, BA2 7AY UK; 2 Faculty of Pharmacy, Quest International University Perak, Ipoh, Perak Malaysia; 3 Genentech, Inc., South San Francisco, California USA; 4 Roche Pharma Research and Early Development, Roche Innovation Center, New York City, New York USA

**Keywords:** surface dissolution, UV imaging, poorly soluble drugs, amorphous formulation, intrinsic dissolution, biorelevant dissolution

## Abstract

The aim of this study is to investigate the dissolution properties of poorly soluble drugs from their pure form and their amorphous formulation under physiological relevant conditions for oral administration based on surface dissolution ultraviolet (UV) imaging. Dissolution of two poorly soluble drugs (cefuroxime axetil and itraconazole) and their amorphous formulations (Zinnat^®^ and Sporanox^®^) was studied with the Sirius Surface Dissolution Imager (SDI). Media simulating the fasted state conditions (compendial and biorelevant) with sequential media/flow rate change were used. The dissolution mechanism of cefuroxime axetil in simulated gastric fluid (SGF), fasted state simulated gastric fluid (FaSSGF) and simulated intestinal fluid (SIF) is predominantly swelling as opposed to the convective flow in fasted state simulated intestinal fluid (FaSSIF-V1), attributed to the effect of mixed micelles. For the itraconazole compact in biorelevant media, a clear upward diffusion of the dissolved itraconazole into the bulk buffer solution is observed. Dissolution of itraconazole from the Sporanox^®^ compact is affected by the polyethylene glycol (PEG) gelling layer and hydroxypropyl methylcellulose (HPMC) matrix, and a steady diffusional dissolution pattern is revealed. A visual representation and a quantitative assessment of dissolution properties of poorly soluble compounds and their amorphous formulation can be obtained with the use of surface dissolution imaging under *in vivo* relevant conditions.

## INTRODUCTION

The amorphous form has attracted increasing interest within the pharmaceutical field because its higher solubility could achieve better dissolution rate and absorption rate and increase the bioavailability of poor water-soluble compounds (1). The solubility increment of amorphous forms over crystalline states depends on the potential energy difference between these physical states (2,3). It was estimated that 10–1600 folds of solubility increment can be achieved by applying the amorphous form (4).

From the physical stability point of view, the drug which is formulated in an amorphous state should be preserved and stabilised to exert its solubility advantage even during the dissolution process (5). There are only a handful of oral pharmaceutical products containing amorphous active pharmaceutical ingredient (API) that have been successfully marketed despite several decades of effort in research and development; examples include cefuroxime axetil (CA) (6), itraconazole (ITR) (7), quinapril (8), etravirine (9), zafirlukast (10) and rosuvastatin (11). The limited commercial success indicates the challenges with the stability of the amorphous formulations (12). One of the issues relating to the stability of the amorphous state is its solution-mediated transformation characteristic. Solution-mediated transformation of amorphous to crystalline state is the conversion of metastable solids such as amorphous solids to the crystalline state when the solids are exposed to a solvent. The transformation to the more thermodynamically stable crystalline state occurs at a higher rate because of the higher mobility in the solution state than in the solid. In drug process development, characterisation of solution-mediated transformations in the amorphous state is important because it describes information on amorphous crystallisation (13).

For poorly water-soluble drugs, the maximum achievable intraluminal drug concentration may limit absorption. However, the intraluminal concentration of a drug is not necessarily limited by its solubility in gastrointestinal fluids (14). Drugs may be in solution at a concentration above their saturation solubility, that is, in a state of supersaturation. A supersaturated drug solution is thermodynamically unstable compared to the equilibrium condition (saturation). Thus, it has the tendency to return to the equilibrium state (lowest chemical potential) by drug precipitation (15). The higher the supersaturation, the more precipitation will take place as the former is the driving force for the latter (16). This higher initial solubility may be sufficient to ensure increased and more rapid absorption for a drug with good permeability such as Biopharmaceutical Classification System (BCS) class 1 and 2 compounds. But, a more thermodynamically stable form may crystallise at any time inside the gastrointestinal (GI) tract and the crystallisation would have a major impact on the product performance *in vivo* (17). The higher dissolution rate and apparent solubility of an amorphous drug usually cause supersaturation during *in vivo* dissolution. Therefore, this may lead to precipitation in the GI tract (as the supersaturation is the driving force for the precipitation) and compromise oral bioavailability (14).

In the GI tract, drug solubility can be enhanced by food and bile components such as bile salts, lecithin and fatty acids. Supersaturation in the intestinal fluid is an important property that can play a significant role in drug absorption. For compounds with poor intrinsic solubility in the intestinal fluid, solubility is often a limiting factor for absorption. For many of these compounds, it may not be possible to enhance the saturation solubility to the extent required such that the whole dose is dissolved in the GI fluid. In this case, creating or maintaining supersaturation in the intestinal fluid can be an effective way to enhance the absorption of these compounds (18).

Surface ultraviolet (UV) dissolution imaging is very useful in characterising active compounds and their formulations, as the captured images illustrate the concentration distribution of drug compounds, which can be translated into the amount and rate of drug dissolution (19–24). The surface UV dissolution imaging is applicable as most pharmaceutical drug substances contain a UV chromophore. The intensity of the measured light is converted to absorbance, creating a high-resolution, real-time 2D absorbance and concentration map of dissolution events within the flow cell which presents a detailed view of the dissolution process occurring on the surface of the drug compact (25). The data can then be processed for measurement of the intrinsic dissolution rate (IDR) of the active compound (pure API or formulation), with the whole process typically taking around 20 min (26,27). The ActiPix™ SDI300 is a multipurpose UV area imaging system which enables quantitative imaging of surface dissolution for various applications such as pure active compound (19,21,23), transdermal patch (20), crystal (22,28), gels (29,30), polymer (31), cocrystal (32–34), excipient shielding (35), salt (36), drug-phospholipid complexes (37), oral strip film (38) and oily liquid (39). Using this system, temporal and high-resolution spatial data from the solid–liquid interface can be observed. Measurement of this dissolution process has been described in length in the literature (19,22,25,29), and it offers insight into surface events such as boundary layer thickness, surface concentration, contour distribution, concentration gradient profiles and surface changes from swelling or gelling. The ActiPix™ SDI300 also supplies special insights into processes occurring in microns to millimetres from the surface, the crucial distance range for recognising dissolution. Moreover, the dissolution medium can be changed easily (for example, from simulated gastric fluid to simulated intestinal fluid) that will reveal the effect of medium (pH and buffer) and hydrodynamics (flow rate) to the precipitation and dissolution of the tested compound. The resulting images with media change setup may increase the understanding of the *in vivo* dissolution process, which may also increase the predictive ability of this dissolution test method. Owing to the fact that the flow-cell volume is small, the time to produce the flow rate changes is relatively short. For example, the flow rate can go from high velocity (4 mL/min) down to no velocity (static, 0 mL/min) almost instantly. Similarly, the low volume also means that less dissolution medium is required, and it is cost-effective when biorelevant media are used. The dissolution setup is very useful for screening potential drug compounds during the pre-formulation stages because the intrinsic dissolution rates can be obtained in less than 20 min compared to 24-h equilibrium IDR using the traditional dissolution system (Wood apparatus).

In this study, two BSC class 2 amorphous compounds (CA and ITR) and their amorphous formulations (Zinnat^®^ and Sporanox^®^, respectively) were used. The study aimed to investigate the dissolution properties of these poorly soluble compounds from their pure form and their amorphous formulation based on surface dissolution UV imaging under physiological relevant conditions for oral administration. The surface of the samples was exposed to media simulating the fasted state conditions: compendial (simulated gastric fluid (SGF) and simulated intestinal fluid (SIF)) and biorelevant (fasted state simulated gastric fluid (FaSSGF) and fasted state simulated intestinal fluid (FaSSIF-V1)) media with sequential media/flow rate change. Biorelevant dissolution media have been used in previous UV dissolution imaging studies (23,40,41) to characterise drug dissolution. To the best of our knowledge, our study is the first study in which an experimental design using media and flow rate change is applied in the UV dissolution imaging.

## MATERIALS AND METHODS

### Materials

Sporanox^®^ capsules (Janssen-Cilag, Ltd., Bucks, UK) and Zinnat^®^ tablets (GlaxoSmithKline, Middlesex, UK) were purchased commercially. ITR standard (98% *w*/*w*) (batch no. 097K1156; St. Louis, MO), CA standard (United States Pharmacopeia (USP) Reference Standard, Lot 09822G; Rockville, MD), amorphous ITR API (intrinsic solubility 0.001 μg/mL) and amorphous CA API (intrinsic solubility 0.4 μg/mL) were provided by Hoffmann-La Roche, Nutley USA.

Glyceryl monooleate (GMO; Rylo M19 Pharma^®^, 99.5% monoglyceride) was a gift from Danisco A/S, Grindsted, Denmark. Egg phosphatidylcholine (Lipoid E PC^®^) was generously donated by Lipoid GmbH, Ludwigshafen, Germany. Sodium oleate (lot number SZBB0110V) and sodium chloride (NaCl) were purchased from Sigma-Aldrich (Dorset, UK). Sodium taurocholate (NaTC, 97% pure) was purchased from Prodotti Chimici e Alimentari S.p.A., Basaluzzo, Italy. The Milli-Q water was obtained from a Nanopure^®^ Diamond UF and UV/UF Water Barnstead System (Thermo Scientific, Dubuque, USA). Buffer components were analytical grade.

### Preparation of Compendial and Biorelevant Media

A fasted state simulating gastric fluid without pepsin (SGF) (42) and fasted state simulating intestinal fluid without pancreatin (SIF) (42) were used to simulate the fasting gastric and intestinal composition, respectively (43,44). Biorelevant media simulating the conditions in the gastric and intestinal environment in the fasted state (FaSSGF and FaSSIF-V1, respectively) were prepared according to the procedure described by Vertzoni *et al.* (45,46).

### Preparation of Compacts

Compacts were prepared using ITR (4 mg) and CA (4 mg) reference standard powder, Sporanox^®^ pellet (4 mg; the hard gelatin Sporanox capsule was removed beforehand) and grounded Zinnat^®^ tablet powder (4 mg; Zinnat^®^ tablet was grounded into fine powder using a mortar and pestle). Samples were transferred into a stainless steel cylinder sample cup (2 mm inner diameter × 2.4 mm height). A Quickset Minor^®^ torque screwdriver (Torqueleader; M.H.H. Engineering Co., Ltd., England) was used to compress the weighted materials at a constant torque pressure of 0.6 Nm for 1 min.

### Surface Dissolution UV Imaging

Sirius SDI (model name: ActiPix™ SDI300; Sirius Analytical Instruments, East Sussex, UK) utilises ActiPix™ UV area imaging technology combined with a laminar flow-through sample holder, an integrated syringe pump and the software. The device consists of a pulsed xenon lamp (PerkinElmer, Waltham, MA) with personal computer interface control box, a remote UV camera sensor with fibre optic cable, a band-pass filter (wavelength 214 nm, 254 nm or 280 nm) and a CADISS-2 dissolution cartridge. The CADISS-2 comprises of a 62-mm rectangular quartz tube, Luer Lock connector flow-cell inlet and outlet as well as a cartridge body. Either the 20-mL and 50-mL BD plastic buffer delivery syringes were used to hold the dissolution medium. Detection was performed at 280 nm (band width 10 nm). The utilised CADISS-2 quartz flow cell had a light path of 4 mm. The imaging system consists of 1280 × 1024 pixels with dimensions of 9 mm^2^ × 7 mm^2^ (total imaging area of 9 mm^2^ × 7 mm^2^), and each pixel can be considered as a conventional single-beam spectrophotometer. Output from the sensor is connected to a computer at a rate of one snapshot every 0.5 s for processing and storage. A syringe pump (RS-232 integrated pump; Maxim Integrated Products, Inc., USA) was used for infusion of dissolution media at constant and changing flow rates, with temperature controlled at 37°C using a Techne DB-2D Dri-Block^®^ digital heater (Staffordshire, UK). Dissolution experiments were performed using ITR and CA reference standard, Sporanox^®^ and Zinnat^®^ compacts, as described below.

### Media and Flow Rate

#### Constant Flow Rate Studies

The flow rates in the ActiPix™ flow cell corresponding to the nominal physiological linear velocity of the fluid in the stomach (2 cm/min) and intestine (1 cm/min) (47) were determined using the following linear relationship: *y* = 7.17*x* − 0.0135 (*R*^2^ = 0.999) (the linear regression of linear velocity of the ActiPix™ flow-through cell [cm/min] *versus* various flow rates [mL/min]). Without taking into account the cross-sectional water loss, the calculated flow rates of the ActiPix™ flow cell are 0.28 mL/min and 0.14 mL/min for the stomach and intestine conditions, respectively (48). Furthermore, to complement the dataset of the flow rate range between 0 and 1.0 mL/min, two bridging flow rates of 0.6 mL/min and 1.0 mL/min were also used for these studies (Table I).

**Table I:** Periods During Which the Compact Was Exposed to the Dissolution Media and the Flow Rate that Each Medium Was Pumped Through the Cell

Medium	Period of each flow rate (min)	Flow rate (mL/min)
ITR and CA pure API compact		
Constant flow rate		
SGF/FaSSGF/SIF^#^/FaSSIF-V1	5 for gastric media	0.14, 0.28, 0.4, 0.6, 0.8, 1
	15 for intestinal media	
Media and flow rate change		
SGF/FaSSGF	0–6	0.8
SIF/FaSSIF-V1	7–24	0.4
Sporanox^®^ pellet compact*		
Constant flow rate		
SGF^#^/FaSSGF/SIF/FaSSIF-V1	5 for gastric media	0.14, 0.28, 0.4, 0.6, 0.8, 1
	15 for intestinal media	
Media and flow rate change		
SGF/FaSSGF	0–6	0.8
SIF/FaSSIF-V1	7–24	0.4

*Experiments with Zinnat^®^ compact were not successful due to the presence of a superdisintegrant in the formulation

^#^Data for Sporanox^®^ pellet compact in SGF with a flow rate of 0.6 mL/min and ITR API compact in SIF with a flow rate of 1.0 mL/min are not available

#### Flow Rate Change Studies

Based on the fact that both the ActiPix™ flow cell and USP apparatus 4 flow-through cell provide unilateral laminar flow and lower turbulence within the flow cell, a one-tenth magnitude scale-down of the corresponding USP apparatus 4 setup was used (49,50). Hence, flow rates of 0.8 mL/min and 0.4 mL/min (reflecting the conditions in the human stomach and intestine, respectively) were used in the experiment with media and flow rate change (Table I).

### Experimental Conditions

The experimental conditions for the studies with surface dissolution UV imaging are described in Table I. Constant flow rate denotes a study using a single dissolution medium under the flow rates of 0.14 mL/min, 0.28 mL/min, 0.4 mL/min, 0.6 mL/min, 0.8 mL/min and 1 mL/min whereas the media and flow rate change denotes a study using, firstly, gastric medium (flow rate 0.8 mL/min) and then intestinal medium (flow rate 0.4 mL/min). Experiments were performed in triplicate unless otherwise indicated, and each run lasted 24 min.

Calibration curves were constructed by flowing drug standard solutions through the dissolution cell at a flow rate of 1 mL/min. Recording of UV images was performed when each of the standard solutions was infused for a period of 5 min. The buffer was infused before and after the series of reference standard solutions for 4 min in order to detect baseline drift. The standard solutions were prepared using CA and ITR reference standard in FaSSGF and SGF, respectively. The concentration range used for both CA and ITR was 5–180 μg/mL; FaSSGF was chosen for CA due to its low degradation in this medium, and SGF was chosen for ITR due to its relatively good solubility in this medium (51). Three UV readings were made at different time points at each concentration, and the average values were used for the calibration plot. The interference caused by the bile salts of FaSSGF or polymer of the solid dispersion was minimum as indicated by the similar absorbance recorded in standard solutions without bile salt and polymers. In each experiment, buffer solution was used to calibrate the spectrophotometer so that any absorbance attributable to the bile salts and polymer as well as particle on the compact surface can be compensated. By setting the absorbance of the blank to zero, an instrumental subtraction measures only the drug absorbance. The collected data were then used to calculate dissolution rates and cumulative amount dissolved of tested compounds in the various media at 1-min intervals, using ActiPix™ D100 software, version 1.5 (York, England) (20,51).

## RESULTS AND DISCUSSION

### Surface Dissolution UV Imaging of Cefuroxime Axetil

An initial dissolution test using Zinnat^®^ (amorphous formulation of CA) compact was not satisfactory because the high loading of super-disintegrant croscarmellose sodium in Zinnat^®^ became a dense layer after being exposed to the dissolution medium (51). This layer caused physical blocking of the light and yielded a large amount of debris that clogged the flow cell, thus preventing further testing of this amorphous formulation of CA (51).

### Single Medium and Constant Flow Rate Study

**Fig. 1:**
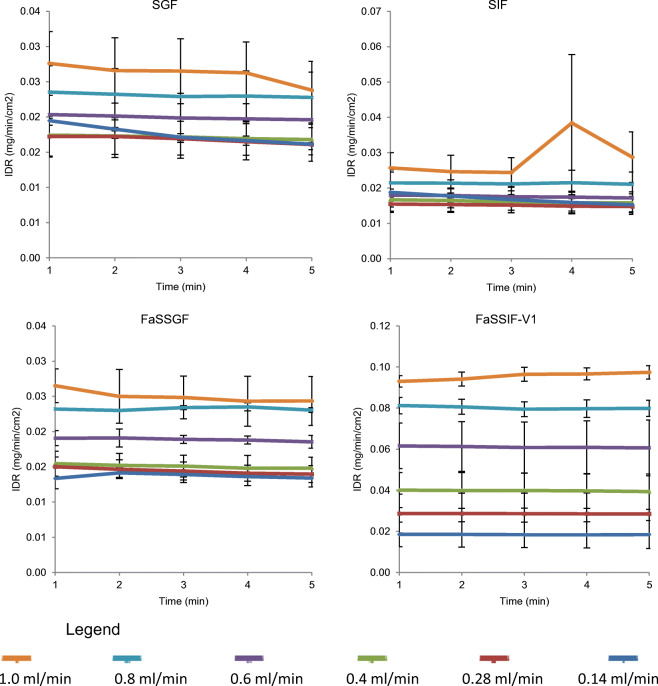
Mean ± SD IDR of CA from its pure API compact in the compendial and biorelevant media (*n* = 3)

The IDR results of CA pure API in compendial and biorelevant media (constant media) *versus* time profiles under various flow rates are presented in Fig. 1.

As observed from the results of the individual constant flow rate studies (flow rate of 1 mL/min, 0.8 mL/min, 0.6 mL/min, 0.4 mL/min, 0.28 mL/min and 0.14 mL/min), the effect of flow rate on the CA IDR profiles is evident (Fig. 1). A clear stepwise IDR reduction in tandem with a flow rate decrease (of each individual constant flow rate experiments) is revealed in FaSSIF-V1.

**Fig. 2:**
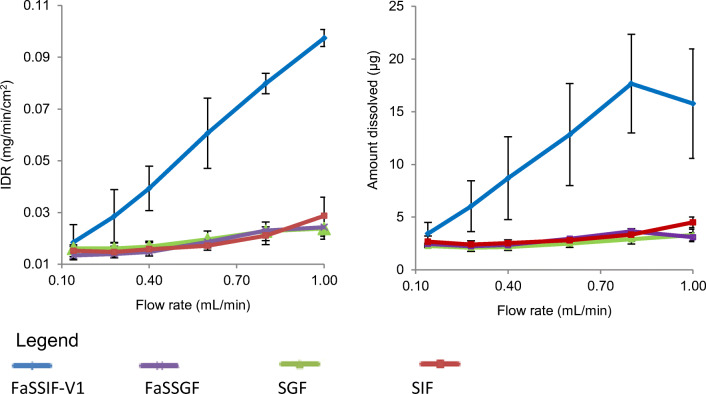
Mean ± SD IDR and amount of CA dissolved at 5 min from its pure API compact in the compendial and biorelevant media (*n* = 3)

A compilation of all measured IDR results *versus* flow rate is shown in Fig. 2. The graph profiles for the studies in SGF, FaSSGF and SIF are almost straight lines and suggest that the dissolution rate is almost constant independent of the flow rate, whereas, with the FaSSIF-V1 profiles, the graph profile shows an apparent gradient which indicates dissolution in convective flow as the main dissolution mechanism. This observation is a good indicator of the effect of mixed micelles that facilitate and enhance the dissolution of poorly water-soluble compounds (52,53). All IDR profiles, regardless of the media used, yielded a plateau IDR *versus* time profile which reveals zero-order release kinetics with a constant drug release rate over a period of time. This indicates that sink conditions for CA dissolution possibly exist in the channel flow cell that would be of importance for the simulation of the *in vivo* conditions (26,48). Comparing specifically with an amorphous furosemide formulation reported elsewhere (41), several similarities were observed. Firstly, a larger area of intense absorbance was observed at the compact surface; secondly, precipitated drug was washed down from the compact surface; and thirdly, absorbant ‘tail’ was observed downstream from the surface of the compact (41).

### Media and Flow Rate Change Study

**Fig. 3:**
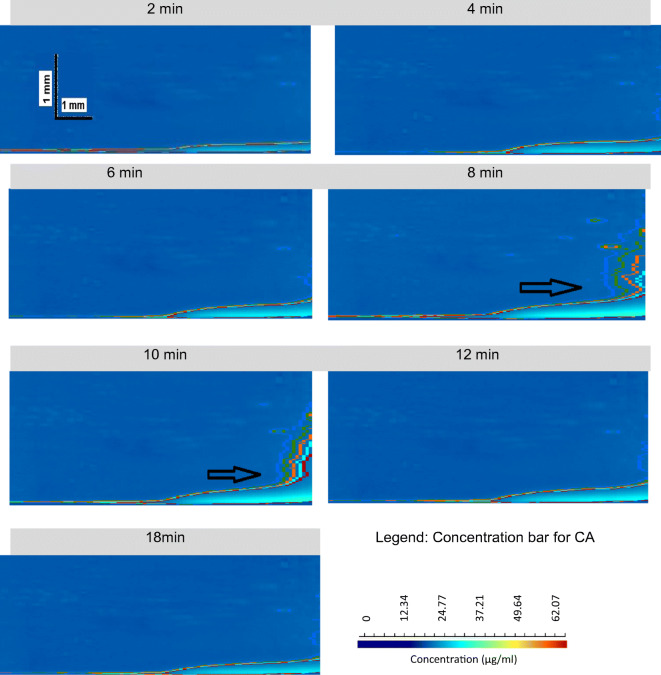
UV concentration maps of CA dissolved from pure API compacts in SGF (0.8 mL/min; 6 min)/ SIF (0.4 mL/min; 18 min) using surface dissolution UV imaging apparatus (Time point: 2, 4, 6, 8, 10, 12 and 18 min).The two horizontal arrows indicate turbulent pattern contour potentially caused by dispersion of the recrystallised particle

Dissolution behaviour of CA was monitored by the surface dissolution UV imaging in SGF (0.8 mL/min; 6 min) followed by SIF (0.4 mL/min; 18 min). The UV images of absorbance maps of dissolved CA at selected time points are shown in Fig. 3. A contour concentration line is presented in the concentration scale bar. All concentrations were lower than 35 μg/mL (within the linear range of the calibration curve). Dissolution occurs as a result of both convection and diffusion, as reported also in other studies (22,23,29,39,54). The absorbance contours reveal that laminar flow conditions are prevailing in the flow cell, while the thickness of the downstream tail varies with the flow rate, similar to what has been shown previously (22,23,29,39,54).

After media and flow rate change, the drug concentration peak at 8 min forms a supersaturated solution and then gradually reduces. An apparent turbulent pattern contour is observed which could probably imply a dispersion of the recrystallised particle at 8 min and 10 min corresponding to the crystal growth post supersaturation (as indicated by the arrow in Fig. 3). The measured dissolution layer thickness at the surface is approximately 0.05 mm to 0.1 mm under both flow rates (0.8 mL/min and 0.4 mL/min), whereas the calculated dissolution layer thickness is 4.34 mm × 10^−3^ mm under 0.8 mL/min and 8.66 mm × 10^−3^ mm under 0.4 mL/min (51).

**Fig. 4:**
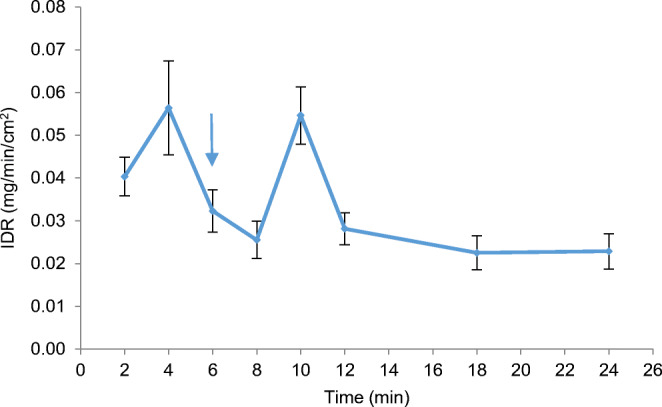
Mean ± SD IDR of CA dissolved from its pure API compact in SGF (0.8 mL/min; 6 min)/SIF (0.4 mL/min; 18 min) (*n* = 2). Blue arrow indicates time with media change

On another note, the measured dissolution layer thickness values using surface UV imaging apparatus are far higher than the calculated dissolution layer thickness. According to the literature (55), this phenomenon suggests that CA dissolved from the surface as aggregate, initially forming a supersaturated solution; this is in agreement with the observed IDR and amount of CA dissolved from the pure API compact with media and flow rate change from SGF (0.8 mL/min) to SIF (0.4 mL/min) (Fig. 4). Figure 4 indicates that there are two supersaturated solutions formed in SGF and SIF before particle precipitation and decrease of IDR. Drug supersaturation, which often precedes precipitation, is conventionally determined *in vitro* through solubility and solution concentration comparison. A recent study conducted using UV-vis imaging, light microscopy and Raman spectroscopy has characterised the piroxicam supersaturation, precipitation and dissolution in an enclosed flow-through casing (15). A study by Sun *et al.* (15) is different as crystalline drug and dissolved drug were inserted instead of the API or formulation that was used in the present study. Our study also reveals that hydrodynamic conditions such as flow rate and flow pattern influence the rate and extent of drug precipitation (15). Furthermore, swelling, crystal growth and/or precipitation at the compact surface can be detected as it has also been shown for the metastable forms of amlodipine besylate where the amorphous salt, the crystalline anhydrate and dihydrate salt forms as well as the amlodipine free base were confirmed (21).

### Surface Dissolution UV Imaging of Itraconazole

#### Single Medium and Constant Flow Rate Study

**Fig. 5:**
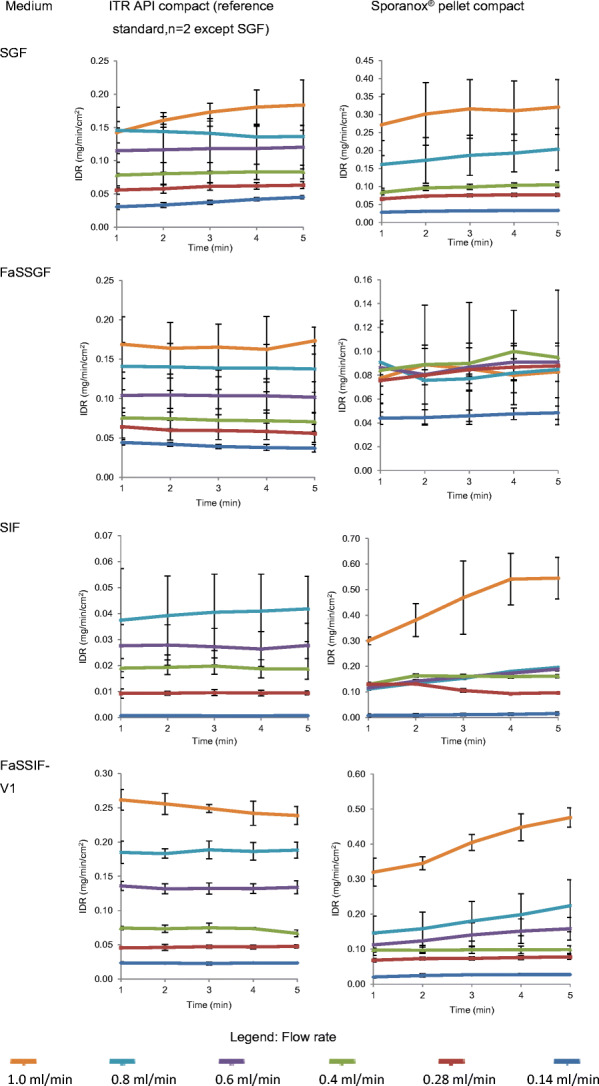
Mean ± SD IDR of ITR from its pure API compact (*n* = 2; *n* = 3 in SGF) and Sporanox^®^ compact (*n* = 3) in the compendial and biorelevant media

**Fig. 6:**
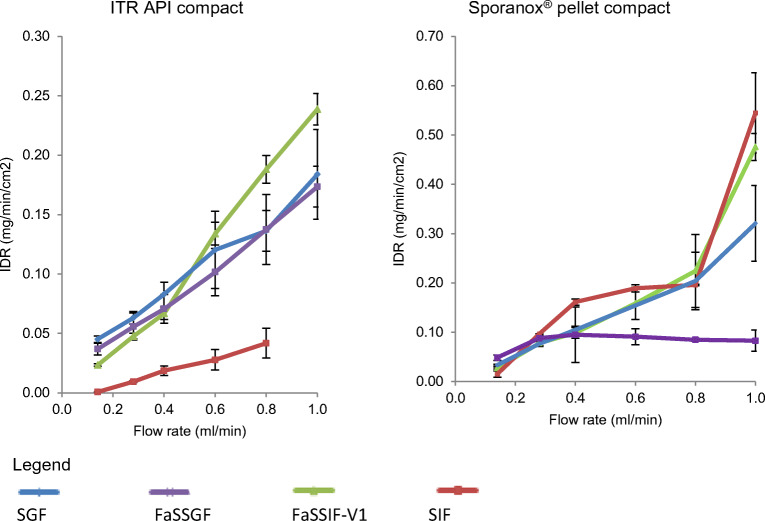
Mean ± SD IDR and amount of ITR dissolved at 5 min from its pure API compact (*n* = 2 in SIF, FaSSGF and FaSSIF-V1) and Sporanox^®^ compact (*n* = 3)

The IDR results of ITR from ITR pure API and Sporanox^®^ compacts in compendial and biorelevant media *versus* time profiles are presented in Fig. 5. A stepwise decrease in dissolution rate corresponding to the stepwise reduction in flow rate is obtained from the imaging data (Fig. 5). This trend is expected based on the convective diffusion theory (56). The key parameter in convective diffusion theory is the concentration at the surface (57). The impact of reducing the flow rate from 1 to 0.14 mL/min on the IDR profiles was evident in ITR pure API in the compendial and biorelevant media and Sporanox^®^ formulation in SGF and FaSSIF-V1. In Sporanox^®^, the IDR profiles in FaSSGF and SIF showed no obvious rank order, probably due to the interference of surface dissolution characterisation in the presence of excipients and polymers, such as hydroxypropyl methylcellulose (HPMC) and polyethylene glycol (PEG). In order to determine the dissolution mechanism of ITR from both compacts, the IDR results were plotted against the flow rate (Fig. 6).

Comparing with analysis using the MRI flow-cell dissolution setup using amorphous felodipine (58), slow erosion of compacted material due to the consolidation of the matrix on compression was also observed in our study. The close resemblance of the flow-cell setup allowed us to indicate that supersaturated drug solution occurred in the proximity of the solid–liquid interface caused by the relatively slow erosion of the compacted materials under the conditions of low convection employed. In agreement with the findings presented by Langham *et al.* (58), nucleation and growth of solid drug particles were driven by the supersaturated solutions.

The dissolution mechanism of ITR from Sporanox^®^ compact (SIF and FaSSIF-V1 at 1 mL/min) showed a steeper curve than from the pure API compact. This is in agreement with the dissolution kinetics of ITR characterised with USP apparatus 4, showing that the dissolution in convective flow is the main process in determining the dissolution of ITR formulated as a solid dispersion–coating pellet (51). The IDR trends in biorelevant media did not reveal the advantage of the surfactants in terms of enhancing the dissolution rate. This is because bile salt has been shown to potentially undergo an acid–base reaction with ITR molecules, leading to slower ITR dissolution (52,53).

The HPMC content in Sporanox^®^ pellet coating is 60% *w*/*w* (ratio of 1:1.5 ITR to HPMC); thus, at this high concentration, the release of ITR is controlled by HPMC. The multiparticulate drug–coated Sporanox^®^ pellet is also coated with the PEG membrane at its outer most layer (7). The rate of ITR dissolution is dependent not only on the thickness of the membrane but also on the composite of the HPMC-ITR solid solution matrix itself which sustains the dissolution (59). Furthermore, HPMC has a gelling property upon hydration which has been identified as a variable affecting drug dissolution (60). Hence, the dissolution of ITR from Sporanox^®^ compact was delayed due to drug particles having to diffuse out from the HPMC matrix before undergoing dissolution.

Due to the poor solubility of ITR in SIF and without the interference of bile salt and lecithin, SIF was able to discriminate Sporanox^®^ formulation (amorphous formulation) and ITR pure API (crystalline compact), with the IDR of ITR from Sporanox^®^ pellet compact in SIF being nearly ten times higher than the one from pure API compact. The results seem to suggest that this experiment setup and conditions could be used to discriminate critical manufacturing variables of such formulations.

#### Media and Flow Rate Change Study

**Fig. 7:**
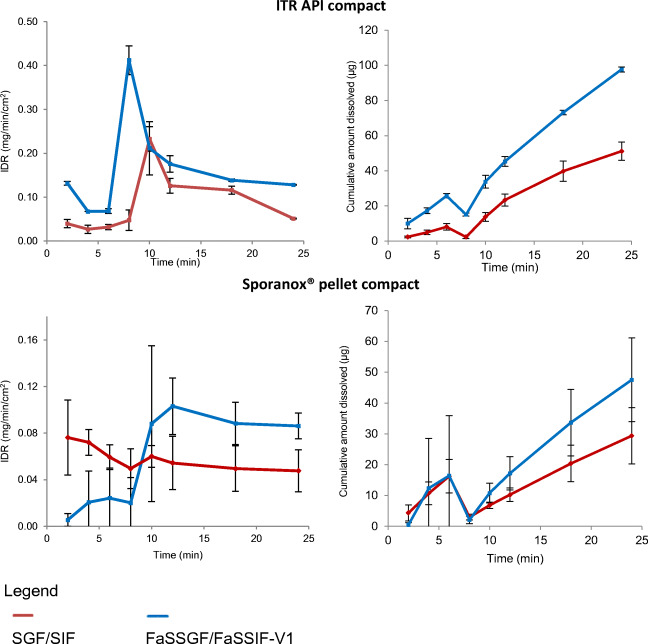
Mean ± SD IDR and amount of ITR dissolved from its pure API and Sporanox^®^ compact in SGF (0.8 mL/min; 6 min)/SIF (0.4 mL/min; 18 min) and in FaSSGF (0.8 mL/min; 6 min)/FaSSIF-V1 (0.4 mL/min; 18 min) (*n* = 3)

Dissolution behaviour of ITR from its pure API and Sporanox^®^ compacts were monitored by the surface UV dissolution apparatus in the sequential change of compendial media (SGF [0.8 mL/min; 6 min] followed by SIF [0.4 mL/min; 18 min]) and in the sequential change of biorelevant media (FaSSGF [0.8 mL/min; 6 min] followed by FaSSIF-V1 [0.4 mL/min; 18 min]). The IDR and cumulative amount of ITR dissolved are presented in Fig. 7.

Compared to the flat and linear profiles in single media (SGF, SIF, FaSSGF and FaSSIF-V1; Fig. 7), the profiles with media change have exhibited their own characteristic gradient. In the dissolution profiles of experiments with media change using surface dissolution UV imaging, a biphasic curve is apparent, with a sudden increment after media change from the simulated gastric medium to the simulated intestinal medium. A higher supersaturation ratio could be observed with the media change from FaSSGF to FaSSIF-V1, due to micellar solubilisation. The same dissolution rate enhancement was observed for the Sporanox^®^ pellet compact in the biorelevant setup (FaSSGF/FaSSIF-V1) but not in the compendial one (SGF/SIF).

The IDR profile of ITR from the pure API compact in SGF/SIF and FaSSGF/FaSSIF-V1 showed that after medium and flow rate changes from simulating gastric condition to simulating intestinal condition, the IDR and amount dissolved increased significantly, forming a transient supersaturated solution of ITR. After 5 min to 8 min, the supersaturation ratio reduced steadily toward the equilibrium dissolution rate of 0.128 mg/min/cm^2^ in FaSSIF-V1 and 0.051 mg/min/cm^2^ in SIF, yielding a total amount of ITR dissolved of 98 μg and 51 μg, respectively. The higher ITR dissolution expected from the amorphous formulation compact was not revealed probably due to the interaction of bile salt (found in FaSSIF-V1) and potassium salt (found in SIF) with PEG (61). The PEG coating used in Sporanox^®^ pellet to prevent agglomeration hinders the ITR dissolution in the surface dissolution UV imaging; this was also shown in the ITR profiles from Sporanox^®^ compacts in the constant biorelevant media (FaSSGF and FaSSIF-V1) study (Fig. 7).

**Fig. 8:**
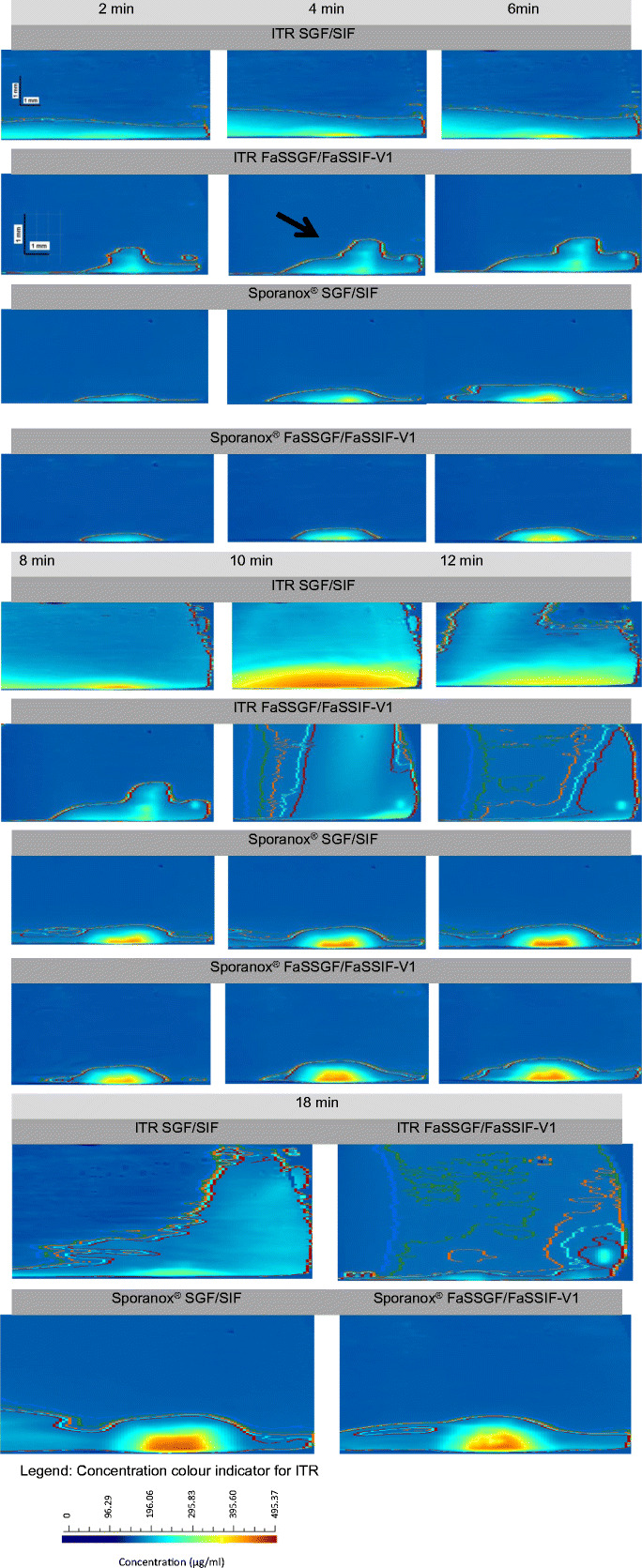
UV concentration maps of ITR dissolved from pure API and Sporanox^®^ pellet compacts in the compendial (SGF [0.8 mL/min; 6 min]/SIF [0.4 mL/min; 18 min]) and biorelevant (FaSSGF [0.8 mL/min; 6 min]/FaSSIF-V1 [0.4 mL/min; 18 min]) media using surface dissolution UV imaging apparatus (time points 2 min, 4 min, 6 min, 8 min, 10 min, 12 min and 18 min)

The concentration curves of ITR dissolved from ITR pure API compact and Sporanox^®^ pellet compact relative to the compact surface axis are presented as concentration contours within the UV absorbance maps of ITR dissolved at different selected time points (Fig. 8). The contours of Sporanox^®^ pellet compacts (Fig. 8) show that density gradients in the proximity of compact surface affect the concentration distribution of the surrounding solutions. Accumulation of dissolved ITR was slightly skewed toward the left part of the contour images for the sample due to a higher density of the HPMC and PEG gel matrix as compared to the solvent. As discussed previously, ITR dissolution from the Sporanox^®^ compact is affected by the PEG gelling layer and HPMC matrix. On the other hand, the contour of ITR pure API compact shows a flat and broad laminar layer. ITR pure API compact in FaSSGF/FaSSIF-V1 forms the least concentrated solution in the dissolution layer compared with the studies in SGF/SIF and also compared to the studies of the Sporanox^®^ compact. There was a clear upward movement of the dissolved ITR into the bulk buffer solution as the downstream distance increases to form a stable dissolution layer. Furthermore, the distorted curve and abnormal shifting of contour lines observed strongly indicate that ITR dissolution is followed by recrystallisation of the ITR particle at the compact surface (black bold arrow in Fig. 8). In the studies using pure API compact with compendial media, although a thicker dissolution layer is observed throughout the whole experiment, the size of the dissolution layer quickly dissipates to the minimum layer at 18 min. In the ITR pure API compact studies with biorelevant media, there was a significant decrease in the dissolution layer after 10 min of the dissolution test that slowly relaxed at a low concentration layer. It could be concluded that the fast supersaturation formed on the surface after media change drastically increased the recrystallisation of ITR on the surfaces of the compacts which could also lead to crystal growth on the surface. A similar setup to examine nilotinib, a protein kinase inhibitor formulated as an amorphous solid dispersion has been reported (27). Compared to the study conducted by Colombo *et al.* (27), solution-mediated phase transformation did not occur to ITR from Sporanox^®^ compact in the beginning of this experiment. ITR from Sporanox^®^ compact precipitated at the later stage of the experiment, confirming that precipitation of an amorphous compound was avoided due to high drug load that prevented matrix swelling during media change (27).

## CONCLUSIONS

Surface dissolution UV imaging methodology that could be used to understand the mechanism of CA and ITR (pure APIs and their amorphous formulations) dissolution was developed in this project. From the surface dissolution UV imaging testing, the UV images of absorbance maps and contour concentration lines of the dissolved CA during the studies with media and flow rate change (SGF/SIF) revealed that CA dissolved from the compact surface as aggregates and formed a supersaturated CA solution that subsequently precipitated out. Similarly, for ITR, the supersaturation of ITR after media change drastically increased the recrystallisation of ITR on the surfaces of the ITR pure API and Sporanox^®^ compacts which could lead to ITR crystal growth. The UV images obtained from these studies provided a visual representation and a qualitative as well as quantitative assessment of the differences in dissolution rates and concentration for the model compounds used.

## Abbreviations

API: Active pharmaceutical ingredient
BCS: Biopharmaceutical Classification System
CA: Cefuroxime axetil
E PC: Egg phosphatidylcholine
FaSSGF: Fasted state simulated gastric fluid
FaSSIF-V1: Fasted state simulated intestinal fluid, version 1
GI: Gastrointestinal
GMO: Glyceryl monooleate
HPMC: Hydroxypropyl methylcellulose
IDR: Intrinsic dissolution rate
ITR: Itraconazole
MRI: Magnetic resonance imaging
NaCl: Sodium chloride
NaTC: Sodium taurocholate
PEG: Polyethylene glycol
SDI: Surface dissolution imaging
SGF: Simulated gastric fluid
SIF: Simulated intestinal fluid
UF: Ultra filtration
UK: United Kingdom
USP: United States Pharmacopeia
UV: Ultraviolet
